# Comparative genomics reveals new single-nucleotide polymorphisms that can assist in identification of adherent-invasive *Escherichia coli*

**DOI:** 10.1038/s41598-018-20843-x

**Published:** 2018-02-09

**Authors:** Carla Camprubí-Font, Mireia Lopez-Siles, Meritxell Ferrer-Guixeras, Laura Niubó-Carulla, Carles Abellà-Ametller, Librado Jesús Garcia-Gil, Margarita Martinez-Medina

**Affiliations:** Laboratory of Molecular Microbiology, Biology Department, Universitat de Girona, Girona, Spain

## Abstract

Adherent-invasive *Escherichia coli* (AIEC) have been involved in Crohn’s disease (CD). Currently, AIEC are identified by time-consuming techniques based on *in vitro* infection of cell lines to determine their ability to adhere to and invade intestinal epithelial cells as well as to survive and replicate within macrophages. Our aim was to find signature sequences that can be used to identify the AIEC pathotype. Comparative genomics was performed between three *E*. *coli* strain pairs, each pair comprised one AIEC and one non-AIEC with identical pulsotype, sequence type and virulence gene carriage. Genetic differences were further analysed in 22 AIEC and 28 non-AIEC isolated from CD patients and controls. The strain pairs showed similar genome structures, and no gene was specific to AIEC. Three single nucleotide polymorphisms displayed different nucleotide distributions between AIEC and non-AIEC, and four correlated with increased adhesion and/or invasion indices. Here, we present a classification algorithm based on the identification of three allelic variants that can predict the AIEC phenotype with 84% accuracy. Our study corroborates the absence of an AIEC-specific genetic marker distributed across all AIEC strains. Nonetheless, point mutations putatively involved in the AIEC phenotype can be used for the molecular identification of the AIEC pathotype.

## Introduction

Crohn’s disease (CD) is an idiopathic chronic inflammatory bowel disease (IBD) involving host genetics, environmental factors and the intestinal microbiome^1^. Alterations of the microbiome known as dysbiosis, in which there is a diminished abundance of beneficial microbes and a subsequent increase in proinflammatory species, have been reported to occur in the gut of CD patients^2,3^. Increased *Escherichia coli* has been repeatedly described in CD^2,4^, and its abundance correlates with disease activity^5^. Boudeau *et al*.^6^ defined a new pathotype of this bacterial species named adherent-invasive *Escherichia coli* (AIEC), which has been associated with CD patients by several independent groups^2–4,7–10^. AIEC possess the ability to adhere to and invade intestinal epithelial cells as well as to survive and replicate inside macrophages^6^. Compared with previously reported pathotypes, AIEC constitute a distinct intestinal pathogenic group of *E*. *coli*. In terms of its virulence genes, AIEC resemble extraintestinal pathogenic *E*. *coli* (ExPEC), which are mostly non-invasive^4,11–13^. Similarly, the AIEC pathotype is clonally diverse, comprising all phylogenetic groups (A, B1, B2, and D), and the latter two are the most abundant in IBD patients^2,14^. In paediatric CD patients, the most prevalent phylogroups are A and D^15^.

Although many of the molecular mechanisms of AIEC pathogenesis have been elucidated^16–20^, there is still no known gene or signature sequence that permits the molecular identification of AIEC strains. At present, identification of the AIEC pathotype relies on time-consuming techniques based on the phenotypic screening of cultured bacteria. Some genes have been more frequently found in AIEC but are also present in some non-AIEC; for instance, the *lpfA* gene has been detected in 68.4–71% of AIEC and 20–28.1% of non-AIEC strains^20,21^. Likewise, *fimH* adhesin is ubiquitous in *E*. *coli*, and although some of its sequence variants have been found to be associated with AIEC, they have also been detected in non-AIEC strains^22–24^. Moreover, point mutations in genes involved in AIEC pathogenicity have been described exclusive to the LF82 strain compared with the commensal K-12 strain. For example, specific gene variants of *ompA*^25^ and *chiA*^26^ have been reported to confer higher invasion and adhesion indices, respectively, to LF82, but confirmation of this finding in a wider collection of strains is needed. Finally, differences between AIEC and non-pathogenic *E*. *coli* may include the expression of specific genes, which has been proposed for type 1 pili^23^.

Several genomic studies have been conducted in attempts to elucidate the characteristics of the AIEC genome and to identify a genetic biomarker. Thirty-four AIEC strains have been sequenced to date, and most belong to the B2 phylogroup^11,12,17,24,27–29^. However, no gene or sequence exclusive to the AIEC pathotype has yet been identified. Genomic studies have confirmed that AIEC resemble ExPEC^17,24,30^. These studies have been useful in the detection of virulence genes that are enriched in CD-isolated AIEC strains relative to those in non-IBD-isolated AIEC strains. In particular, the *fyuA* and *ibeA* genes, which are involved in the production of siderophores and invasiveness, respectively, were found in more than 70% of CD-AIEC strains and were absent from non-IBD-AIEC strains^15^. In addition, genomic studies have also shown that virulence genes such as *pduC*, which is involved in invasiveness and persistence, are more frequent in AIEC than in non-AIEC (50% and 20%, respectively)^17^.

Difficulties in discovering AIEC-specific traits have been probably due to the fact that the first studies compared AIEC and non-AIEC strains phylogenetically distant and the differences between these strains are related to their phylogenetic origin rather than the AIEC phenotype^11,12^. Recently, by comparing AIEC and non-AIEC strains of the same phylogroup, Desilets *et al*.^24^ found three genomic regions present in all B2-phylogroup AIEC strains and absent from AIEC strains of other phylogroups and commensal strains of any phylogenetic origin (including B2). However, whether these regions are specific to B2-AIEC strains only or also present in other pathogenic groups that share the same phylogenetic origin, such as B2 ExPEC strains is unknown. Desphande *et al*.^30^ described 29 diagnostic single-nucleotide polymorphisms (SNPs) that cause either synonymous or non-synonymous amino acid changes as a signature sequence that differentiates a group of B2-pathogenic strains comprising 4 AIEC, 3 ExPEC, 47 uropathogenic *E*. *coli* and 1 avian pathogenic *E*. *coli* strains from other *E*. *coli* strains present in the NCBI database. However, no specific characteristic that discriminates the AIEC pathotype was found. Finally, O’Brien *et al*.^27^ reduced gene content variability by conducting genomic analysis of a set of B2-phylogroup *E*. *coli* strains with identical sequence type (ST95), thereby decreasing the likelihood of detecting differential genetic elements delimited by the phylogenetic background. Nonetheless, the evaluation of gene prevalence and base composition of core genes did not result in the identification of an AIEC-specific biomarker or even in the identification of a marker common to most of the AIEC strains examined in that study.

Taken together, these studies demonstrate that gene content is mostly associated with the phylogenetic origin of individual strains rather than with their AIEC pathotype. Moreover, genes enriched in AIEC are also present in other *E*. *coli* pathotypes and even in non-pathogenic *E*. *coli*. However, differences in the expression of particular genes according to pathotype have been suggested^23,31^. Therefore, we hypothesise that point mutations in core genes and/or differences in gene expression may be responsible for the AIEC phenotype.

In this study, we focused on differences in gene content and SNPs of AIEC and non-AIEC strains isolated from the human intestine. With the aim of finding an AIEC-specific biomarker, we sequenced the genomes of six *E*. *coli* strains comprising three pairs of AIEC/non-AIEC strains. Each pair of strains was isolated from the same patient and had an identical phylogroup origin, sequence type, virulence gene profile, and pulsed-field gel electrophoresis (PFGE) fingerprint. Our hypothesis was that comparing genetically close strain pairs increases the likelihood of finding specific genetic elements characteristic of the AIEC phenotype. Moreover, we explored the usefulness of the genetic differences found as molecular markers for AIEC identification by studying them in a diverse collection of AIEC and non-AIEC strains and we have finally proposed an algorithm for AIEC classification.

## Results

### Characteristics of strain pairs

Three AIEC strains and three corresponding non-AIEC counterparts that shared identical PFGE patterns (Fig. S1), sequence types, phylogenetic origins and virulence genes (Table 1) with the AIEC strains were selected for genome sequencing in the present study. As expected, the AIEC strains presented higher adhesion and invasion indices than did their non-AIEC counterparts. However, the non-AIEC strains also had the capacity to survive and replicate inside J774 murine macrophages but not in human THP-1 macrophages, with the exception of the ECG28 strain.

**Table 1:** Characteristics of the three sequenced AIEC/non-AIEC strain pairs.

Strain	Phylogroup	Serotype	ST^a^	Virulence genes	Adhesion index^bφ^	Invasion index^c ϕ^	Intramacrophage replication index in J774^dΦ^	Intramacrophage replication index in THP-1^d¥^
AIEC17	D	ONT:HNT	569	*fimC*, *mat*, *ompA*, *ea/I*, *sitA*, *sitD_ch*, *irp2*, *fyuA*, *chuA*, *vat*, *ibeA*, *kpsMTII*, *neuC*, *traT*, csgA, *fimH*, *chiA*, *gipA*, *pduC*	21.6 ± 17.5	0.266 ± 0.055	1053 ± 75	213 ± 60
ECG28					0.6 ± 0.3	0.005 ± 0.001	774 ± 129	228 ± 68
AIEC01	B2	O6:H1	73	*focG*, *mat*, *iha*, *ompA*, *pic*, *sitA*, *sitD_ch*, *irp2*, *iucD*, *iutA*, *fyuA*, *vat*, *pks*, *kpsMTII*, *traT*, *fimH*, *chiA*, *gipA*, *chuA*	15.9 ± 9.3	0.284 ± 0.106	1567 ± 1060	173 ± 99
ECG11					1.1 ± 0.8	0.004 ± 0.002	716 ± 315	74 ± 29
AIEC07	B1	O22:H7	3232	*fimC*, *mat*, *csgA*, *ompA*, *fimH*, *lpfA*_154_, *gipA*, *chuA*, *fyuA*	20.0 ± 13.4	0.565 ± 0.392	1693 ± 297	189 ± 71
ECG04					1.8 ± 0.7	0.036 ± 0.029	527 ± 194	77 ± 17

^a^Sequence type. ^b^Number of bacteria per I-407 cell. ^c^Percentage of intracellular bacteria after 1 h gentamicin treatment relative to the inoculum. The percentages of reduction of invasion for AIEC17, AIEC01 and AIEC07 were 99.8%, 99.4% and 99.8%, respectively, in the presence of cytochalasin D and 90.4%, 99% and 95%, respectively, in the presence of colchicine. ^d^Percentage of intracellular bacteria present at 24 h post-infection relative to the number of intracellular bacteria present after 1 h of gentamicin treatment. Results of control strains LF82 and K-12 strains respectively: ^φ^25.66 ± 15.7 and 0.70 ± 0.02. ^ϕ^2.26 ± 1.349 and 0.019 ± 0.020. ^Φ^777 ± 304.8 and 11 ± 5. ^¥^121 ± 59 and 10 ± 7. Strains with an adhesion index ≥ 1, an invasion index ≥ 0.1% and an intramacrophage replication index ≥ 100% were classified as AIEC.

Similar total genome sizes ranging from 4,825 to 5,213 Kb were obtained for the AIEC and non-AIEC strains (Table S1), and no significant structural differences were found between the strain pairs (Fig. S2). One inversion was detected in all pairwise comparisons, but the inversed regions were not homologous among the three strain pairs.

Genes that were previously associated with AIEC were searched in the genomes of the six strains. The *gipA*, *chuA* and *fyuA* genes were present in the three AIEC strains and in their non-AIEC counterparts (Table 1). Other genes were not present in any strain (*afaC*) or present only in a single strain pair (*lpfA*_154_, *pduC* and *ibeA*). Similar results were obtained for the analysis of the predicted amino acid sequences encoded by the *fimH*, *ompA* and *chiA* genes, for which the same variants were found within a strain pair (Table S2). Clustered regulary interspaced palindromic repeats (CRISPR) analysis was also performed, and equal profiles were obtained for the pairs AIEC17-ECG28 and AIEC07-ECG04; no confirmed CRISPR was recognized in AIEC01 or in ECG11.

### Comparative genomics of AIEC/non-AIEC strain pairs

The genomes of the three AIEC/non-AIEC strain pairs were compared within pairs to identify gene content differences and SNPs that could be implicated in the AIEC phenotype.

#### Evaluation of gene content dissimilarities

**Figure 1:**
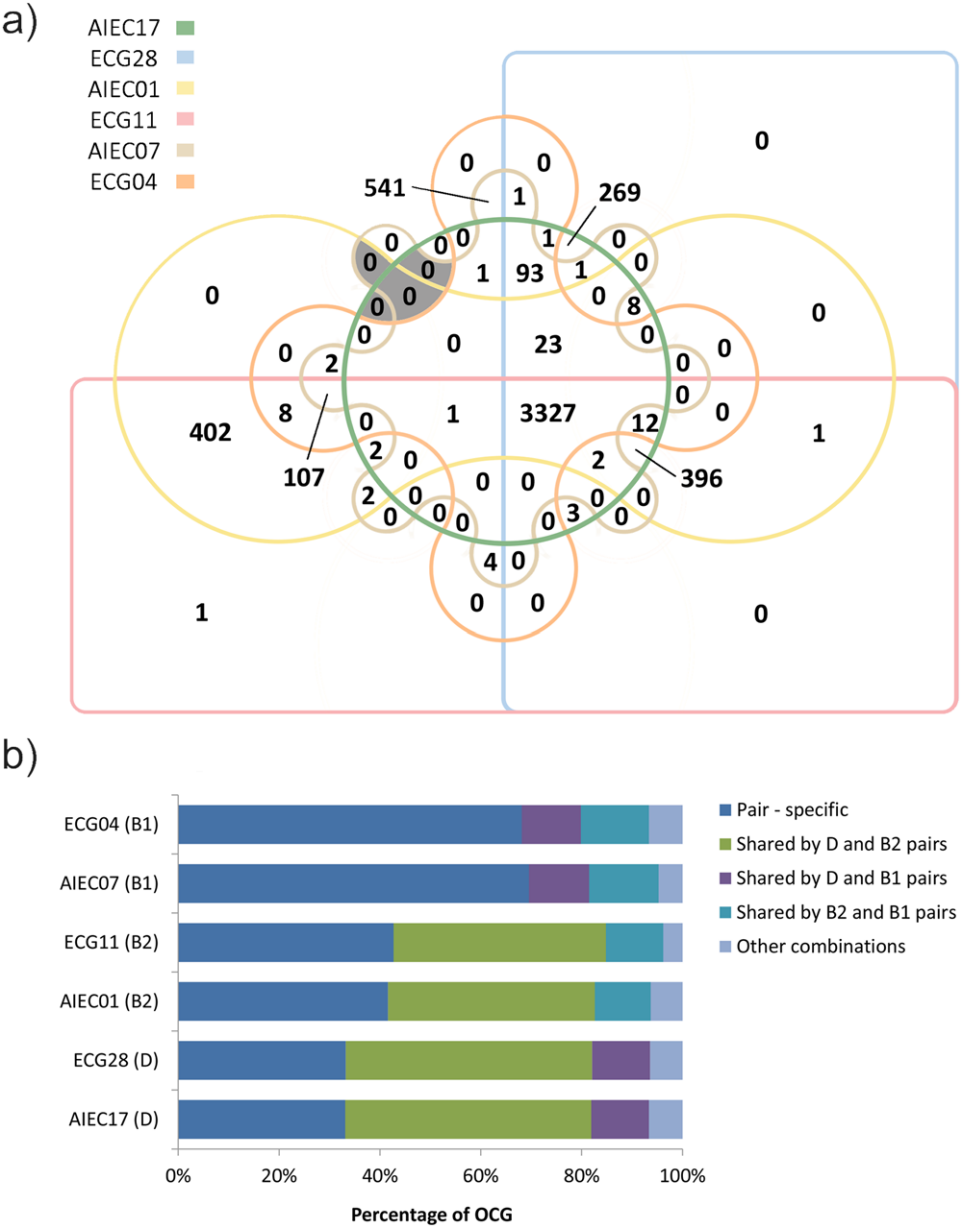
Genome similarities among the six strains and within each pair by analysis of orthologous clusters of genes (OCG) analysis. (**a**) Edward’s Venn diagram indicating the number of OCG. The shadowed areas correspond to clusters exclusively shared between at least two AIEC strains. (**b**) Percentage of OCG between phylogenetically distinct strain pairs and among AIEC strains. Percentages are calculated in relation to the number of variable OCG for each strain. Those OCG that are not present in all six strains are considered variable. Other combinations include gene clusters shared by 5 strains or 3 strains from two or three different phylogroups. There were no common OCG among AIEC strains.

Homologous protein-encoding sequences were identified. A total of 5208 orthologous clusters of genes were obtained: 3327 (63.9%) clusters were common to the six strains (Fig. 1a). These genes represented 80%, 77% and 81% of the genomes of the AIEC17-ECG28, AIEC01-ECG11 and AIEC07-ECG04 pairs, respectively. The strain pairs belonging to the B2 and D phylogroups shared a higher proportion of orthologous clusters of genes (9.2–9.6% of their genomes) than the B1 strains shared with B2 (2.5–2.6%) or D (2.2–2.3%) (Fig. 1b). The high similarity between strain pairs was also evidenced in terms of gene content; because strains within a pair shared more than 99.2% of orthologous clusters of genes.

None of the clusters were shared by the three AIEC strains, not even exclusively by two of them (Fig. 1a), indicating the absence of AIEC-specific genes that were present in all AIEC strains. However, within each strain pair, exclusive AIEC genes were identified (Table S3). AIEC17 contains two genes encoding uncharacterized proteins (YgiZ and YeeW), one gene encoding a cyanate transporter and a gene encoding a TraR family protein that is involved in a quorum-sensing process^32^. In the case of AIEC01, 33 of the genes present in its genome were absent from the genome of ECG11. Of those 33 genes, 20 encode proteins of unknown or generic function, six are related to transmembrane transport, four are implicated in transcriptional regulation, and three contribute to flagellum assembly. Finally, compared with ECG04, AIEC07 harbours three proteins of unknown function (one of which is an *E*. *coli* uropathogenic-specific protein), one protein related to intracellular iron transport (TonB), and the autotransporter UpaH. The latter protein mediates biofilm formation in the uropathogenic strain CFT073^33^. Biofilm formation is also a phenotypic trait of AIEC strains^34^.

#### Detection of AIEC-associated SNPs

In our approach to identifying AIEC-associated SNPs, we focused our attention on the nucleotide positions that varied between the strains of a pair and were also located in homologous sequences of an AIEC reference genome (UM146 strain). A total of 286 polymorphisms were found (Data set S1); the majority (213) of the SNPs were located in gene encoding sequences (Table 2). Of these SNPs, 60 were selected for resequencing, and only 20 of them were confirmed by Sanger sequencing. These SNPs were located in 11 genes, its location and function is presented in Table 3.

**Table 2:** Number of SNPs in the sequenced AIEC/non-AIEC strain pairs.

	AIEC17-ECG28	AIEC01-ECG11	AIEC07-ECG04
Total SNPs	51	126	109
Total SNPs in genes	40	91	82
Selected SNPs*^1^	20 (7)	10 (5)	30 (19)
Confirmed SNPs*^2^	4 (4)	7 (3)	9 (4)
SNPs studied in a strain collection*^3^	4 (4)	4 (2)	8 (3)

*The number of genes in which the SNPs are located is indicated in parenthesis.

^1^SNPs that conform to the following criteria: (I) cause a non-synonymous amino acid change; (II) are not located at the end of a contig; and (III) were validated bioinformatically by ClustalW.

^2^Validated by Sanger method.

^3^Confirmed SNPs that were not strain-specific (and four strain-specific SNPs for validation).

Of note, 14 of these SNPs (70%) were found to present overlapping peaks in the Sanger chromatograms (Table 3). We hypothesised that i) strains with ambiguous bases may possess more than one copy of the relevant gene in their genomes or ii) there is intraclonal variability in the polymorphic site. To identify which of these possibilities gave rise to the ambiguous SNPs, we first performed BLASTn^35^ searches in the strains’ genomes and the genome of the reference AIEC strain to search for duplicate genes. Next, we analysed next-generation sequencing (NGS) reads using Tablet^36^ to confirm our observations. Duplicated genes showed a unique or main nucleotide in the SNP that differed from the nucleotide present in the other gene copy (this was the case for the SNPs found in the E3-E4_4.3, E3-E4_4.4 and E3-E4_4.7 genes). Genes that were not duplicated showed a single result in BLASTn and two different nucleotides that were almost equally frequent in the sequencing reads (this was the case for the SNPs found in the E5-E6_3.16 = 3.22 and E5-E6_3.17 genes). Therefore, we suggest that the overlapping peaks were not due to technical artefacts.

**Table 3:** Location of the confirmed SNPs, nucleotide variants and gene functions.

Gene ID	Strain pair where identified	SNP location in AIEC genome (Contig: position)	Nucleotide variant*^1^ (AIEC/non-AIEC)	Protein name	Protein family	Gene Ontology
E1-E2_3.4	AIEC17 vs ECG28	8:204059	C/T	GntR family transcriptional regulator	PF00392; PF07702	GO:0003677; GO:0003700; GO:0006351
E1-E2_3.6		105:325	C/T	Phage protein	PF06174	
E1-E2_3.7		3:50414	T/C	Serine peptidase DegQ	PF13365; PF13180; PF00595	GO:0004252
E1-E2_5		51:69	G/T	Vitamin B12 transporter BtuB	PF07715	GO:0009279; GO:0015235; GO:0006811; GO:0046872; GO:0046930; GO:0015288; GO:0004872
E3-E4_4.3	AIEC01 vs ECG11	3:167, 173, 209	C/Y, Y/Y, T/K	Putative uncharacterised protein	PF06174	
E3-E4_4.4		84:1126	R/R	dGTPase	PF00350	GO:0005525
E3-E4_4.7		80:920, 932, 1013	S/S, S/S, Y/Y	Chemotaxis protein	PF13990	
E5-E6_3.1	AIEC07 vs ECG04	3:6356	A/C	FimH	PF00419; PF09160	GO:0007155; GO:0009289
E5-E6_3.12		83:442	A/G	Succinyl –CoA ligase subunit beta	PF08442	GO:0005524; GO:0000287; GO:0030145; GO:0004775; GO:0006099
E5-E6_3.16 = 3.22		51:418, 544, 545, 633, 646, 650	Y/Y, S/S, R/R M/M, Y/Y, S/S	Enterobacterial Ail/Lom family protein	PF06316	GO:0009279; GO:0016021
E5-E6_3.17		62:583	R/R	Putative prophage component	PF00877	

*^1^A:adenine; C:cytosine; G:guanine; K:guanine or thymine; M:adenine or cytosine; R:adenine or guanine; S:guanine or cytosine; T:thymine; W:adenine or thymine; Y:cytosine or thymine.

### Distribution of SNPs in an AIEC/non-AIEC strain collection

We studied the variability of nucleotides within the identified SNPs in a collection of AIEC and non-AIEC strains to validate or refute the hypothesis that Confirmed SNPs represent putative molecular signatures for the specific identification of the AIEC pathotype. Sixteen SNPs were studied in 22 AIEC and 28 non-AIEC strains isolated from healthy subjects and CD patients belonging to several phylogroups (A (n = 9), B1 (n = 7), B2 (n = 28), D (n = 5) and atypical (n = 1)) (Table S4). We selected only those Confirmed SNPs that were not strain-specific as assessed *in silico* because strain-specific SNPs would be uninformative for the identification of AIEC. Four SNPs considered strain-specific *in silico* (E1-E2_3.4, E1-E2_5, E1-E2_3.7, and E5-E6_3.1) were also analysed to confirm that they were strain-specific.

Interestingly, some nucleotide variants occurred more frequently in AIEC strains than in non-AIEC strains (Table 4). In particular, variants with thymidine in SNP E3-E4_4.3(2) were found only in AIEC, whereas those containing cytosine were more frequent within non-AIEC. Similar results were obtained when the analysis was restricted to B2 phylogroup strains (p = 0.037); in this phylogroup, AIEC strains (n = 6) were again the only ones presenting thymidine, and cytosine was also more prevalent in non-AIEC than in AIEC strains (n = 10 and n = 7, respectively). This gene was present in all the strains studied, and the SNP variants were not associated with the phylogroup origin of the strains but only with the AIEC phenotype. Another intriguing SNP was present in gene E3-E4_4.4 in which 42.86% of non-AIEC strains presented guanine, whereas less than 10% of the AIEC strains presented this variant. However, in this case, not all strains harboured the gene, as occurs for the LF82 strain. Finally, a guanine in SNP E5-E6_3.16 = 3.22(2) that presented a high variability within the strain collection was more frequently found in AIEC strains, whereas a cytosine at this position was associated with non-AIEC strains. If only B2 strains are considered, the cytosine variant is found exclusively in non-AIEC strains (n = 5), whereas the guanine variant is specific to AIEC strains (n = 4) (p = 0.007). Therefore, this variant could be of interest as a biomarker for B2-phylogroup AIEC strains. However, the percentages of strains that present these two variants are low (41.7% of B2 non-AIEC and 30.8% of B2 AIEC).

**Table 4:** Prevalence of genes encompassing SNPs in a collection of AIEC/non-AIEC strains and the frequency of particular nucleotide variants with respect to AIEC phenotype and phylogroup origin of the strains. Only SNPs presenting statistically significant differences regarding pathotype are presented. Values are given in percentages with respect to the total number of AIEC or non-AIEC strains. Statistically significant differences for each variant are presented in bold type. *For phylogroup analysis, the atypical non-AIEC strain was discarded.

SNP ID	PCR amplification		SNP base vs AIEC phenotype				SNP base vs phylogroup				
	AIEC (n = 22)	non-AIEC (n = 28)	Ntd (N strains)	AIEC	non-AIEC	p-value	A (n = 9)	B1 (n = 7)	B2 (n = 28)	D (n = 5)	p-value
E3-E4_4.3(2)	100	100	C (33)	**45**.**45**	**82**.**14**	**<0**.**001**	77.78	71.43	60.71	60.00	0.667
			T (9)	**40**.**91**	**0**.**00**		22.22	0.00	21.43	20.00	
			Y (8)	13.64	17.86		0.00	28.57	17.86	20.00	
E3-E4_4.4	77.28	71.43	A (5)	13.64	7.14	**0**.**010**	0.00	14.29	14.29	0.00	0.636
			G (14)	**9**.**09**	**42**.**86**		33.33	28.57	32.14	0.00	
			R (18)	**54**.**55**	**21**.**43**		33.33	14.29	42.86	40.00	
E5-E6_3.16 = 3.22(2)	90.91	89.28	C (14)	**13**.**64**	**39**.**29**	**0**.**012**	44.45	28.57	17.86	40.00	**0**.**026**
			G (8)	**31**.**82**	**3**.**57**		22.22	0.00	14.29	40.00	
			T (13)	36.36	17.86		**22**.**22**	**0**.**00**	**39**.**29**	**0**.**00**	
			S (5)	9.09	10.71		**0**.**00**	**42**.**86**	**7**.**14**	**0**.**00**	
			K (3)	0.00	10.71		0.00	0.00	10.71	0.00	
			Y (2)	0.00	7.14		11.11	14.29	0.00	0.00	

A:adenine; C:cytosine; G:guanine; K:guanine or thymine; M:adenine or cytosine; R:adenine or guanine; S:guanine or cytosine; T:thymine; W:adenine or thymine; Y:cytosine or thymine.

### SNPs in relation to adhesion and invasion capacity

**Figure 2:**
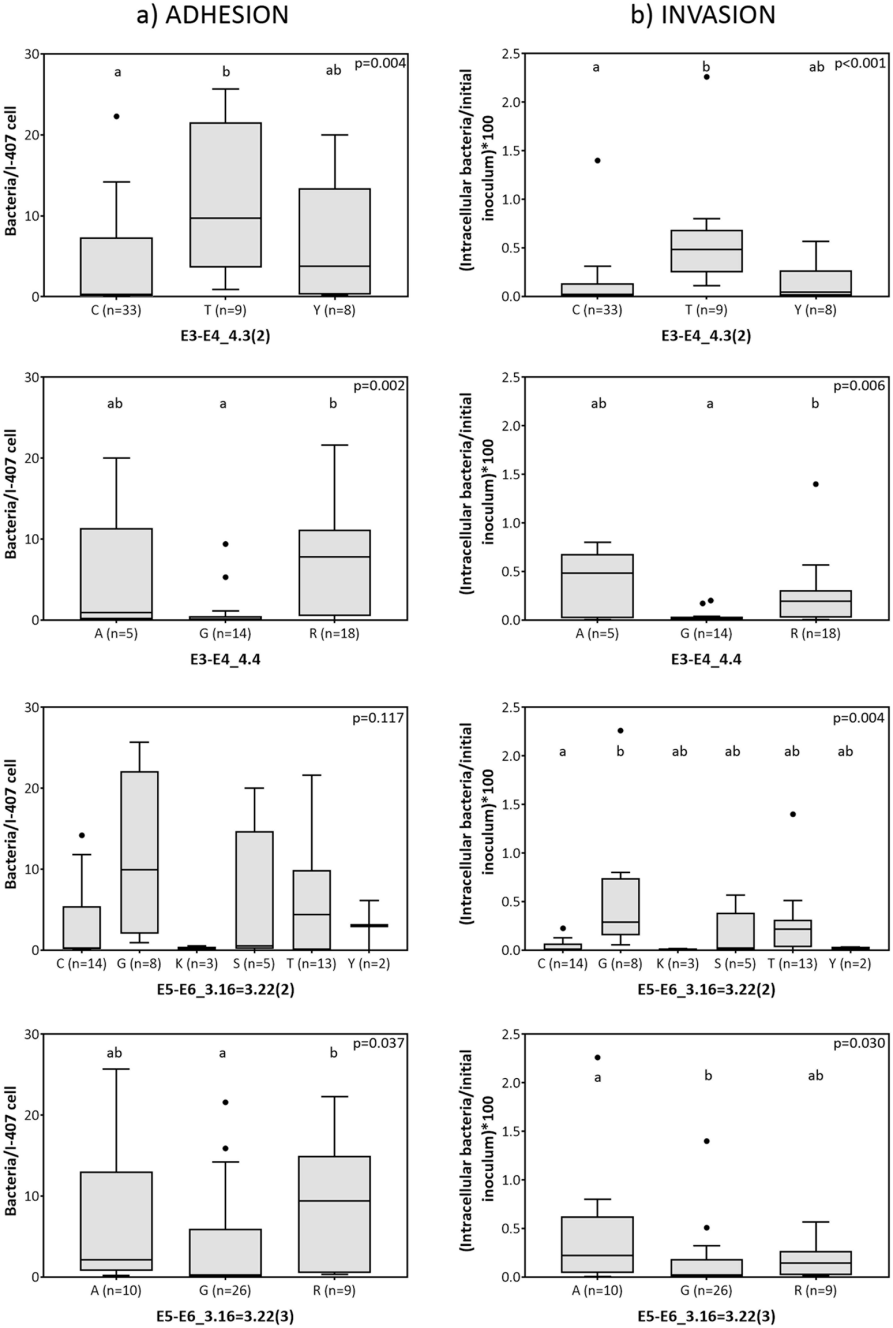
Adhesion (**a**) and invasion (**b**) abilities of the strains according to specific nucleotide variants of SNPs. Only SNPs associated with significant differences (p < 0.05 using the Mann-Whitney U-test) in the adhesion or invasion abilities of variants are shown. Homogeneous subgroups (p > 0.05) within each panel are indicated by the same superscripts. The median of the data is indicated by the horizontal line in each box, boxes cover the 25% and 75% quantiles, and bars show the 10% and 90% quantiles. Outliers are marked as dots.

As expected, strains carrying SNP variants associated with the AIEC pathotype showed increased adhesion and invasion indices (Fig. 2). The single exception was SNP E5-E6_3.16 = 3.22(2), in which increased adhesion did not reach statistical significance. In turn, strains with guanine in SNP E3-E4_4.4 showed the lowest adhesion and invasion indices. An additional polymorphism, SNP E5-E6_3.16 = 3.22(3), showed significant differences; strains with adenine in this SNP displayed increased invasive ability.

SNPs E5-E6_3.16 = 3.22(2) and E5-E6_3.16 = 3.22(3) are consecutive and result in the same amino acid change. To visualise their effects on amino acid sequence, we focused on the possible nucleotide combinations found across our *E*. *coli* strain collection. The combinations of the two changing positions lead to the possibility of 13 SNP variants that can be translated in 6 different amino acids. As expected, the guanine-adenine combination was associated with the highest invasion values (0.63% ± 0.76). Statistically significant differences according to pathotype were observed when the guanine-adenine combination (n = 7) was compared with the cytosine-guanine combination (n = 11) (p = 0.009). The former leads to a basic amino acid at pH = 7 (serine), whereas the latter encodes a neutral amino acid (alanine) that may affect the function of the protein.

### Usefulness of SNPs as molecular signatures for AIEC screening

The use of a binary logistic regression model revealed two SNPs that are predictive of AIEC phenotype (Table 5). The SNP in E3-E4_4.4 can classify the strains as AIEC or non-AIEC with 73% global success, *E*. *coli* strains with a nucleotide base other than guanine at this position have a 65.2% of probability of exhibiting the AIEC phenotype, whereas those strains presenting guanine have only a 14.3% probability of exhibiting the AIEC phenotype. In the case of SNP E5-E6_3.16 = 3.22(2), global success was similar (68.9%), but only 35% of the AIEC strains were correctly classified.

**Table 5:** Binary logistic regression model for the SNPs associated with the AIEC pathotype. The equation variables, the risk of being AIEC (odds ratio), the p-value of the regression model and the percentage of successfully classified strains are indicated.

	Equation variables				Observed	Predicted			
	B	p-value	Odds ratio	95% CI		Non-AIEC	AIEC	% Correct	Global %
Not to have G in E3-E4_4.4	2.420	0.006	11.250	2.004–63.168	Non-AIEC	12	8	60.0	73.0
Constant	−1.792	0.019	0.167		AIEC	2	15	88.2	
To have G in E5-E6_3.16 = 3.22(2)	2.559	0.023	12.923	1.430–116.785	Non-AIEC	24	1	96.0	68.9
Constant	−0.613	0.075	0.542		AIEC	13	7	35.0	

**Figure 3:**
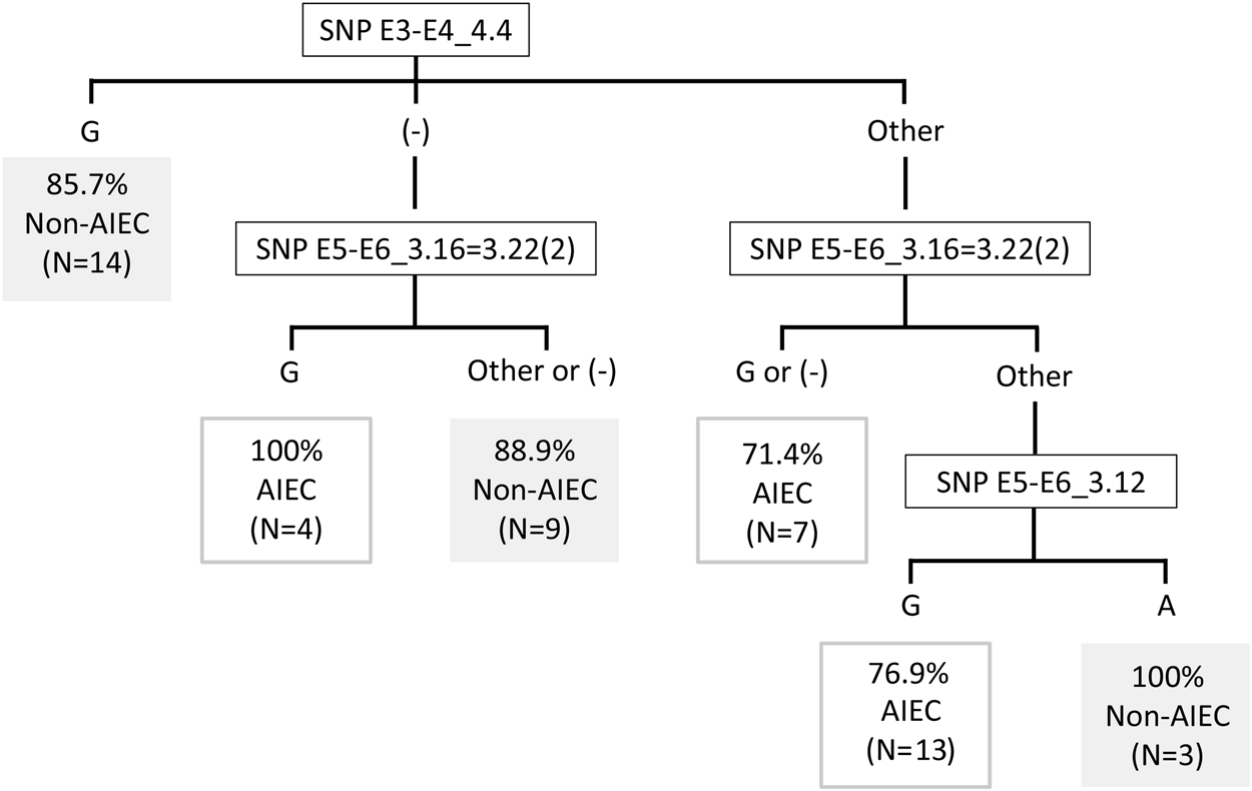
Classification algorithm for AIEC identification. Percentages represent the proportion of strains that are correctly predicted as AIEC or non-AIEC based on the result for each SNP combination. The number of total strains corresponding to each condition is indicated. (−): no amplification; other: a nucleotide different from guanine (G) or overlapping peaks.

Although the global success in AIEC prediction based on the SNP in E3-E4_4.4 was high, 42.9% of non-AIEC strains were misclassified as AIEC. To improve prediction specificity, we designed a classification algorithm based on the identification of the nucleotides present in three SNPs (Fig. 3). In this algorithm, the variant in SNP E3-E4_4.4 is first determined, and those strains containing a guanine are classified as non-AIEC with a percentage of success of 85.7%. Strains with another result in SNP E3-E4_4.4 (adenine, overlapping peak of adenine and guanine (R) or gene absence) are then analysed for the SNP E5-E6_3.16 = 3.22(2). The combined results obtained for both SNPs can classify the strains with a percentage of success that ranges from 71.4% to 100%, with the exception of isolates that present nucleotides different from guanine in both genes, which remain unclassifiable. For that reason, we included a third SNP (E5-E6_3.12). Despite not being more frequently found in AIEC considering the whole strain collection (39.3% of non-AIEC and 22.7% of AIEC presented adenine, whereas 60.7% of non-AIEC and 77.3% of AIEC presented guanine; p = 0.174), this SNP was useful in classifying this particular group of strains (50% of non-AIEC and 0% of AIEC strains presented adenine, whereas 50% of non-AIEC and 100% of AIEC strains presented guanine; p = 0.036). Overall, the classification algorithm displays 82.1% specificity, 86.4% sensitivity and 84.0% accuracy within our strain collection.

These results collectively suggest that although we were unable to find molecular signatures specific to AIEC and present in all AIEC, our approach identifies some genes with polymorphisms that may represent an advantage or disadvantage for the adhesion and invasion abilities of *E*. *coli* and may thus be involved in the AIEC phenotype. Moreover, we have designed a novel molecular strategy based on the identification of the nucleotides present in three polymorphic sites that can be implemented as a tool for AIEC identification.

## Discussion

The AIEC pathotype is of interest due to its association with gut inflammation in CD patients^2–4,7–10^. At present, AIEC pathotype identification is conducted by phenotypic screening of cultured bacteria, which is an extremely time-consuming technique. Thus, the existence of a molecular tool for the specific detection of AIECs would be of great significance in facilitating, for instance, studies of AIEC distribution that define pathotype reservoirs and transmission paths.

In this study, comparative genomics between AIEC and non-AIEC strains that are considered clones with respect to their PFGE patterns has been performed for the first time. In contrast to previous comparative genomics studies, our methodology precludes the detection of genome variations that are inherent, for example, in the phylogenetic origin of the strains. This approach may increase the chance of identifying molecular signatures that are specific to AIEC. In fact, previous difficulties in discovering the distinctive features of AIEC strains could occur because non-phylogenetically related AIEC and non-AIEC strains have usually been compared^17,24^ and most AIEC studied belonged to a particular phylogroup^27,30^. We chose to sequence strain pairs that belong to different phylogroups (B1, B2 and D) and studied the distribution of the differences found in a collection that included isolates of different phylogenetic origin to determine whether they were universal among AIEC strains and absent from non-AIEC strains.

No significant differences in genome structure or even in gene content were found between AIEC and non-AIEC strains, confirming their close identity by PFGE. No gene was present in at least two AIEC strains and absent from all non-AIEC strains. However, small differences in gene content were observed between strains of the same pair. Nevertheless, this result should be confirmed because it could be a consequence of incomplete genome assembly (fragmented genes will not be found). In general, our results support the idea that the AIEC phenotype is not determined by the presence or absence of a particular gene, as O’Brien *et al*.^27^.

Our analysis revealed no association between the presence of previously AIEC-associated genes (*lpfA*_154_, *gipA*, *pduC*, *fyuA*, *afaC*, *chuA* and *ibeA*)^15–17,20,21,37,38^ and the AIEC phenotype, in concordance with previous observations^17,27^. A similar situation was found with respect to the genetic variants of *fimH*, *ompA* and *chiA*. Although specific changes in the amino acid sequences of proteins encoded by these genes have been associated with higher adhesion/invasion capacity^23,25,26^, we did not observe such differences between the isolates of our strain pairs. Therefore, differences in the sequences of these genes might not determine the pathotype of the strains. However, as Dreux *et al*.^23^ suggested, gene expression should be evaluated in depth because it could also be involved in the determination of the phenotype.

In a further attempt to explain the observed phenotypic differences between pairs, we sought to identify SNPs that were differentially present in AIEC strains and their non-AIEC counterparts. The rate of occurrence of such SNPs that were further validated by Sanger sequencing was low. This result can be explained by the accumulation of small errors during library construction and sequencing caused by the imperfect fidelity of DNA polymerases and the intrinsic error rate of the sequencing platform, as well as errors derived from the parameters used for the assembly of the reads^39,40^. Occasionally, there is variability and the nucleotide that appears in the consensus assembled contig does not represent all the reads. Although resequencing by the Sanger method is not frequently used to validate NGS data, our results are consistent with the results of a previous work^41^. In light of the evidence, we highly recommend confirmation of NGS data, especially in SNP research. Another intriguing result was the presence of genes with intraclonal polymorphisms. Bacterial cultures grown overnight may represent genetically heterogeneous populations^42^.

The distribution of the polymorphisms in our strain collection indicated the absence of a particular nucleotide from any of the SNP positions that was present in all AIEC strains and differed from the base found in that position in non-AIEC strains. However, we found SNP positions that presented differences not only in the distribution of nucleotide variants according to pathotype (E3-E4_4.3(2), E3-E4_4.4 and E5-E6_3.16 = 3.22(2)) but also in their association with adhesion and invasion indices (E3-E4_4.3(2), E3-E4_4.4, E5-E6_3.16 = 3.22(2), and E5-E6_3.16 = 3.22(3)). From a functional point of view, the E5-E6_3.16 = 3.22 gene encodes a protein of the enterobacterial Ail/Lom family. This protein family includes outer membrane proteins involved in bacterial virulence, such as OmpX (in *E*.*coli* and *Enterobacter cloacae*)^43^ and PagC (in *Salmonella typhimurium*)^44^. These proteins play roles in cell adhesion and intramacrophage survival, respectively. The SNP-containing gene E3-E4_4.4 encodes a dGTPase that is a member of the dynamin-like protein family (PF00350), whose function in bacteria is poorly understood. Finally, E3E4_4.3 is of unknown function, yet it shares 97.2% (74.5% coverage) amino acid sequence homology with the hypothetical protein yeeT. The low sequence homology of these genes with genes in the currently available databases makes it difficult to identify the proteins encoded by these genes with confidence. Therefore, the creation of isogenic mutants will be needed to further understand the biological function of these proteins and demonstrate the effects of their possible amino acid variations.

Interestingly, two of the identified SNPs were adequate for the prediction of the AIEC phenotype as determined by the binary logistic regression model. Moreover, we present here a classification algorithm that combines three SNPs, which allows the classification of phylogenetically diverse *E*. *coli* isolates with a high accuracy rate. Using this algorithm, 84% of our *E*. *coli* strains were correctly classified as AIEC or non-AIEC. Of note, the AIEC 12–1 ti12 and non-AIEC 12–2 ti13 strains sequenced by O’Brien *et al*.^27^, both strains isolated from the same patient and with the same ST (ST127), are correctly classified with the molecular tool presented here. The application of this tool may assist in overcoming the problem of AIEC identification; however, further confirmation of its validity in additional geographically distant and phylogenetically diverse AIEC strains is necessary. To our knowledge, only one reported study has searched for SNPs in the entire genome of AIEC using pathogenic and non-pathogenic *E*. *coli*^30^. The authors reported 29 diagnostic SNPs that can differentiate a group of AIEC, but these SNPs are also present in ExPEC, specifically in uropathogenic *E*. *coli* and avian pathogenic *E*. *coli*. Unfortunately, no specific polymorphism that exclusively differentiates AIEC strains from other pathogenic *E*. *coli* strains has yet been described. Therefore, studying E3-E4_4.4, E5-E6_3.16 = 3.22(2) and E5-E6_3.12 polymorphic sites in other pathogenic *E*. *coli* is necessary to confirm the specificity of the tool.

In conclusion, even though no genetic element could be designated specific for AIEC classification, our data reveal three SNPs that can be implemented in AIEC identification. In contrast to classical cell culture infection-based assays, this approach represents a rapid and standardisable method for detecting AIEC from *E*. *coli* isolates. Although this tool does not correctly classify all *E*. *coli* strains, its accuracy is very high (84%), and no comparable molecular tools currently exist. Further studies are needed to demonstrate or rule out the role that the variants reported in this study play in the AIEC phenotype. Taken together, the results of this study provide meaningful information that contributes to our understanding of AIEC genomics.

## Methods

### Strains selection and characterisation

Three *E*. *coli* strain pairs isolated in a previous study^2^, each consisting of one AIEC and one non-AIEC of identical pulsotype and belonging to a distinct phylogroup (B1, B2 or D), were selected. The study from which the AIEC strains were obtained was approved on May 22, 2006 by the Ethics Committee of Clinical Investigation of the Hospital Josep Trueta of Girona. AIEC07/ECG04 pair was isolated from the ileum of a control patient while AIEC17/ECG28 and AIEC01/ECG11 pairs were isolated from the colon and ileum of an I-CD patient, respectively. Information on the patients from whom all the strains used in this study were isolated is presented in Table S5. The main characteristics of the sequenced strains are shown in Table 1. The selection criteria, multilocus sequence typing (MLST), PFGE protocol and the resulting dendrograms are presented in the Supplementary Materials and Fig. S1. Phenotypic characterisation of the selected strains was performed to determine adhesion and invasion of the intestine-407 epithelial cell line (I-407 (American Type Culture Collection (ATCC) CCL-6)) as well as survival and replication within two macrophage cell lines (J774A.1 (ATCC TIB-67) and THP-1 (ATCC TIB-202) from mouse and human, respectively) as detailed in the Supplementary Materials. Strains with an adhesion index ≥ 1, an invasion index ≥ 0.1 (that was reduced by 90% to 99.9% when the microfilament inhibitor cytochalasin D and the microtubule inhibitor colchicine were added), and a replication index ≥ 100% in J774 and THP-1 were classified as AIEC strains in the present study.

A selection of genes previously associated with the AIEC phenotype either due to their higher prevalence in the pathotype (genes *lpfA*_154_^17,21^, *gipA*^20^, *chuA*^17^, *fyuA*^45^, *afaC*^37^, *pduC*^17^ and *ibeA*^16^) or due to the presence of amino acid variants relevant to the pathotype (FimH^23^, OmpA^25^ and ChiA^26^) was examined using BLASTn^35^ and ClustalW alignment^46^.

### Genomic DNA extraction and sequencing

Genomic DNA was extracted from bacterial cells cultured overnight in Luria-Bertani (LB) culture broth using the Wizard® Genomic DNA Purification kit (Promega) according to the manufacturer’s instructions; samples were treated with RNase A provided with the kit. DNA purity was determined using a NanoDrop ND-100 spectrophotometer (NanoDrop Technologies), DNA quantity was measured using a Qubit® 2.0 Fluorometer (Life Technologies), and DNA integrity and RNA elimination were examined on agarose gels. Unique bands of approximately 23 Kb were identified in agarose gels. The 260/280 ratio of the DNA preparations ranged from 1.8 to 2, and the quantity of DNA ranged from 15 to 30 µg, indicating sufficient quality of the genomic DNA for genome sequencing. Two sequencing platforms, Illumina HiSeq and PacBio Biosciences (Supplementary Materials), were used.

### *De novo* genome assembly

Draft genomes were assembled *de novo* (combining both platforms) using SPAdes software (ABL)^47^ and annotated using the BG7 bacterial genome annotation pipeline^48^. To assign gene function, the hit with the lowest E-value obtained after analysis against the UniProt database was chosen. The draft genomes have been deposited in the European Nucleotide Archive under the accession numbers ERS1456453 (AIEC17), ERS1456454 (ECG28), ERS1456455 (AIEC01), ERS1456456 (ECG11), ERS1456457 (AIEC07) and ERS1456458 (ECG04).

### Comparative genomics of strain pairs

#### Differences in genome structure and gene content

Mauve 2.3^49^ was used to identify structural rearrangements and inversions throughout the strain’s genome. CRISPRFinder (http://crispr.i2bc.paris-saclay.fr/)^50^ was used to study CRISPR. To find gene content differences, BLASTP comparison (E-value cutoff 1e-5) and Markov clustering (inflation factor 2.0) were performed by ORTHOVENN^51^ using protein sequences. A correction with local BLASTn^35^ was performed to recruit genes located at the beginning or end of contigs.

#### Single-nucleotide polymorphisms in coding regions

The Harvest suite for rapid-core genome alignment^52^ was used to detect SNPs between strains of the same pair. To maximize sensitivity, the best annotated AIEC genome at the moment of the analysis, UM146 (NC_017632.1)^28^, was used as a reference genome. Therefore, only those genes homologous to UM146 were considered for the analysis. For the purpose of this study, SNPs chosen for examination were those that caused non-synonymous amino acid changes in coding regions and were not present in highly variable regions or at the ends of contigs. These SNPs are referred to as “Selected SNPs”.

The Selected SNPs were validated by Sanger resequencing. Using Primer3, primers were designed to flank at least 100 bp up- and downstream of the SNP position to achieve good sequence quality for assigning nucleotides at the position of the SNP. Care was taken to design primer sets that target the conserved regions of several AIEC and non-AIEC strains. All primers were further analysed with NetPrimer to select the optimal primer pairs. The validated polymorphisms are referred to as “Confirmed SNPs”. The primers and the polymerase chain reaction (PCR) conditions used are presented in Table S6.

Of note, we found some SNPs with ambiguous nucleotide peaks (called “SNPs with overlapping peaks”) that represent a mixture of two nucleotides at a given position. The possible cause of these ambiguous nucleotide peaks was analysed *in silico* using a combination of BLASTn^35^ gene searches and inspection of reads through Tablet^36^ (Supplementary Materials). The next step in the selection of SNPs was the identification of strain-specific SNPs; these SNPs were discarded from the analysis (Supplementary Materials).

Genes harbouring Confirmed SNPs were classified using the Gene Ontology Consortium^53^ and Pfam databases^54^.

### Distribution of SNPs among a collection of strains

To analyse the putative application of the Confirmed SNPs as biomarkers for the specific identification of AIEC, a total of 16 SNPs present in 9 genes were screened in a wider strain collection of 22 AIEC and 28 non-AIEC strains^2^ (Tables S4 and S5) by Sanger sequencing.

### Statistical analysis

The differences in the distribution of nucleotides present in each polymorphic site between pathotype and phylogroups were calculated using the Χ^2^ test. The non-parametric Kruskal-Wallis test was used to compare the mean adhesion and invasion indices among more than two nucleotide variants, and the Mann-Whitney U-test was performed to analyse pairwise comparisons. Binary logistic regression was employed as a model to predict AIEC pathotype according to the nucleotide present in a particular SNP position. A p-value ≤ 0.05 was considered statistically significant in all cases.

### Data availability

All data generated or analysed during this study are included in this published article (and its Supplementary Information files).

## Electronic supplementary material

Supplementary Materials

Supplementary Data Set S1

## References

[1] Sartor RB (2006). Mechanisms of disease: pathogenesis of Crohn’s disease and ulcerative colitis. Nat Clin Pr. Gastroenterol Hepatol.

[2] Martinez-Medina M (2009). Molecular diversity of *Escherichia coli* in the human gut: New ecological evidence supporting the role of adherent-invasive *E*. *coli* (AIEC) in Crohn’s disease. Inflamm. Bowel Dis..

[3] Darfeuille-Michaud A (2004). High prevalence of adherent-invasive *Escherichia coli* associated with ileal mucosa in Crohn’s disease. Gastroenterology.

[4] Baumgart M (2007). Culture independent analysis of ileal mucosa reveals a selective increase in invasive *Escherichia coli* of novel phylogeny relative to depletion of *Clostridiales* in Crohn’s disease involving the ileum. ISME J..

[5] Lopez-Siles M (2014). Mucosa-associated *Faecalibacterium prausnitzii* and *Escherichia coli* co-abundance can distinguish Irritable Bowel Syndrome and Inflammatory Bowel Disease phenotypes. Int. J. Med. Microbiol..

[6] Boudeau J, Glasser AL, Masseret E, Joly B, Darfeuille-Michaud A (1999). Invasive ability of an *Escherichia coli* strain isolated from the ileal mucosa of a patient with Crohn’s disease. Infect. Immun..

[7] Martin HM (2004). Enhanced *Escherichia coli* adherence and invasion in Crohn’s disease and colon cancer. Gastroenterology.

[8] Sasaki, M. *et al*. Invasive *Escherichia coli* are a feature of Crohn’s disease. *Lab*. *Investig*. **87** (2007). 10.1038/labinvest.3700661 17660846

[9] Eaves-Pyles T (2008). *Escherichia coli* isolated from a Crohn’s disease patient adheres, invades, and induces inflammatory responses in polarized intestinal epithelial cells. Int. J. Med. Microbiol..

[10] Dogan B (2013). Multidrug resistance is common in *Escherichia coli* associated with Ileal Crohn’s Disease. Inflamm. Bowel Dis..

[11] Miquel S (2010). Complete genome sequence of crohn’s disease-associated adherent-invasive *E*. *coli* strain LF82. PLoS One.

[12] Nash JH (2010). Genome sequence of adherent-invasive *Escherichia coli* and comparative genomic analysis with other *E*. *coli* pathotypes. BMC Genomics.

[13] Martinez-Medina M (2009). Similarity and divergence among adherent-invasive *Escherichia coli* and extraintestinal pathogenic *E*. *coli* strains. J. Clin. Microbiol..

[14] Kotlowski R, Bernstein CN, Sepehri S, Krause DO (2007). High prevalence of *Escherichia coli* belonging to the B2 + D phylogenetic group in inflammatory bowel disease. Gut.

[15] Conte MP (2014). Adherent-invasive *Escherichia coli* (AIEC) in pediatric Crohn’s disease patients: phenotypic and genetic pathogenic features. BMC Res. Notes.

[16] Cieza RJ, Hu J, Ross BN, Sbrana E, Torres AG (2015). The IbeA invasin of adherent-invasive *Escherichia coli* mediates interaction with intestinal epithelia and macrophages. Infect. Immun..

[17] Dogan B (2014). Inflammation-associated Adherent-invasive *Escherichia coli* Are enriched in pathways for use of propanediol and iron and M-cell. Inflamm. Bowel Dis..

[18] Gibold, L. *et al*. The Vat-AIEC protease promotes crossing of the intestinal mucus layer by Crohn’s disease-associated *Escherichia coli*. *Cell*. *Microbiol*. **18**(5), 617–31, 10.1111/cmi.12539 (2016). 10.1111/cmi.12539 26499863

[19] Martinez-medina M, Garcia-gil LJ (2014). Escherichia coli in chronic inflammatory bowel diseases: An update on adherent invasive Escherichia coli pathogenicity..

[20] Vazeille E (2016). GipA factor supports colonization of Peyer’s Patches by Crohn’s Disease-associated *Escherichia Coli*. Inflamm. Bowel Dis..

[21] Chassaing B (2011). Crohn disease-associated adherent-invasive *E*. *coli* bacteria target mouse and human Peyer’s patches via long polar fimbriae. J. Clin. Invest..

[22] Iebba V (2012). Microevolution in *fimH* gene of mucosa-associated *Escherichia coli* strains isolated from pediatric patients with inflammatory bowel disease. Infect. Immun..

[23] Dreux N (2013). Point mutations in FimH adhesin of Crohn’s Disease-associated Adherent-invasive *Escherichia coli* enhance intestinal inflammatory response. PLoS Pathog..

[24] Desilets M (2015). Genome-based definition of an Inflammatory Bowel Disease-associated Adherent-invasive *Escherichia coli* pathovar. Inflamm. Bowel Dis..

[25] Rolhion N (2010). Abnormally expressed ER stress response chaperone Gp96 in CD favours adherent-invasive *Escherichia coli* invasion. Gut.

[26] Low D (2013). Chitin-binding domains of *Escherichia coli* ChiA mediate interactions with intestinal epithelial cells in mice with colitis. Gastroenterology.

[27] O’Brien, C. L. *et al*. Comparative genomics of Crohn’s disease-associated adherent-invasive *Escherichia coli. Gut*. **66**(8), 1382–1389. 10.1136/gutjnl-2015–311059 (2017). 10.1136/gutjnl-2015-311059 27196580

[28] Krause DO, Little AC, Dowd SE, Bernstein CN (2011). Complete genome sequence of adherent invasive *Escherichia coli* UM146 isolated from ileal crohn’s disease biopsy tissue. J. Bacteriol..

[29] Clarke DJ (2011). Complete genome sequence of the crohn’s disease-associated adherent-invasive *Escherichia coli* strain HM605. J. Bacteriol..

[30] Deshpande, N. P., Wilkins, M. R., Mitchell, H. M. & Kaakoush, N. O. Novel genetic markers define a subgroup of pathogenic *Escherichia coli* strains belonging to the B2 phylogenetic group. *FEMS Microbiol*. *Lett*. **362**(22), 1–7, 10.1093/femsle/fnv193 (2015). 10.1093/femsle/fnv193 26459886

[31] Rolhion N, Carvalho FA, Darfeuille-Michaud A (2007). OmpC and the σE regulatory pathway are involved in adhesion and invasion of the Crohn’s disease-associated *Escherichia coli* strain LF82. Mol. Microbiol..

[32] Vannini A (2002). The crystal structure of the quorum sensing protein TraR bound to its autoinducer and target DNA. EMBO J..

[33] Allsopp LP (2010). UpaH is a newly identified autotransporter protein that contributes to biofilm formation and bladder colonization by uropathogenic *Escherichia coli* CFT073. Infect. Immun..

[34] Martinez-Medina M (2009). Biofilm formation as a novel phenotypic feature of adherent-invasive *Escherichia coli* (AIEC). BMC Microbiol..

[35] Altschul SF (1997). Gapped BLAST and PSI-BLAST: a new generation of protein database search programs. Nucleic Acids Res..

[36] Milne I (2013). Using Tablet for visual exploration of second-generation sequencing data. Brief. Bioinform..

[37] Prorok-Hamon M (2014). Colonic mucosa-associated diffusely adherent *afaC*+ *Escherichia coli* expressing *lpfA* and *pks* are increased in inflammatory bowel disease and colon cancer. Gut.

[38] Céspedes S (2017). Genetic diversity and virulence determinants of *Escherichia coli* strains isolated from patients with Crohn’s Disease in Spain and Chile. Front. Microbiol..

[39] Olson ND (2015). Best practices for evaluating single nucleotide variant calling methods for microbial genomics. Front. Genet..

[40] McElroy K, Thomas T, Luciani F (2014). Deep sequencing of evolving pathogen populations: applications, errors, and bioinformatic solutions. Microb. Inform. Exp..

[41] den Bakker HC (2010). Comparative genomics of the bacterial genus *Listeria*: Genome evolution is characterized by limited gene acquisition and limited gene loss. BMC Genomics.

[42] Herring CD (2006). Comparative genome sequencing of *Escherichia coli* allows observation of bacterial evolution on a laboratory timescale. Nat. Genet..

[43] Meng X (2016). Virulence characteristics of extraintestinal pathogenic *Escherichia coli* deletion of gene encoding the outer membrane protein X. J. Vet. Med. Sci.

[44] Pulkkinen WS, Miller SI (1991). A *Salmonella typhimurium* virulence protein is similar to a *Yersinia enterocolitica* invasion protein and a bacteriophage lambda outer membrane protein. J. Bacteriol..

[45] Spurbeck RR (2012). *Escherichia coli* isolates that carry *vat*, *fyuA*, *chuA*, and *yfcV* efficiently colonize the urinary tract. Infect. Immun..

[46] Thompson JD, Higgins DG, Gibson TJ (1994). CLUSTAL W: improving the sensitivity of progressive multiple sequence alignment through sequence weighting, position-specific gap penalties and weight matrix choice. Nucleic Acids Res..

[47] Bankevich A (2012). SPAdes: a new genome assembly algorithm and its applications to single-cell sequencing. J. Comput. Biol..

[48] Pareja-Tobes P, Manrique M, Pareja-Tobes E, Pareja E, Tobes R (2012). BG7: A new approach for bacterial genome annotation designed for Next Generation Sequencing Data. PLoS One.

[49] Darling AE, Mau B, Perna NT (2010). ProgressiveMauve: multiple genome alignment with gene gain, loss and rearrangement. PLoS One.

[50] Grissa I, Vergnaud G, Pourcel C (2007). CRISPRFinder: a web tool to identify clustered regularly interspaced short palindromic repeats. Nucleic Acids Res..

[51] Wang Y, Coleman-Derr D, Chen G, Gu YQ (2015). OrthoVenn: A web server for genome wide comparison and annotation of orthologous clusters across multiple species. Nucleic Acids Res..

[52] Treangen TJ, Ondov BD, Koren S, Phillippy AM (2014). The Harvest suite for rapid core-genome alignment and visualization of thousands of intraspecific microbial genomes. Genome Biol..

[53] Gene Ontology Consortium, T. G. O (2015). Gene Ontology Consortium: going forward. Nucleic Acids Res..

[54] Finn RD (2016). The Pfam protein families database: towards a more sustainable future. Nucleic Acids Res..

