# Supplementary material for: Comparative genomics reveals new single-nucleotide polymorphisms that can assist in identification of adherent-invasive Escherichia coli
Source: Sci Rep. 2018 Feb 9;8:2695. doi: 10.1038/s41598-018-20843-x (PMC5807354; doi:10.1038/s41598-018-20843-x)
Supplement: Supplementary file 1 — Supplementary Materials [file 41598_2018_20843_MOESM1_ESM.pdf]

Comparative genomics reveals new single-nucleotide polymorphisms that can assist in identification of adherent-invasive *Escherichia coli*

Carla Camprubí-Font[1], Mireia Lopez-Siles[1], Meritxell Ferrer-Guixeras[1], Laura Niubó-Carulla[1], Carles Abellà-Ametller[1], Librado Jesús Garcia-Gil[1] and Margarita Martinez-Medina[1*]

[1]Laboratory of Molecular Microbiology, Biology Department, Universitat de Girona, Girona, Spain.

*marga.martinez@udg.edu.

SUPPLEMENTARY MATERIALS

Selection of strains

Three AIEC strains isolated in a previous study[1] were selected based on the following criteria: i) possessing different phylogenetic origins, ii) displaying high adhesion (>15.9 bacteria/I-407 cell) and invasion (>0.266% of inoculum surviving after 1 h of gentamicin treatment) indices in Intestine-407 (I-407; ATCC CCL-6) cells and iii) possessing an ExPEC-like genotype. The main characteristics of these strains are shown in Table 1. Non-AIEC strains with pulsotypes identical to those of each selected AIEC were searched in the *E. coli* collection obtained for each patient in the same previous study[1].

Multilocus sequence typing

Multilocus sequence typing (MLST) was performed *in silico* by querying the sequences of 7 housekeeping genes (*adk, fumC, gyrB, icd, mdh, purA,* and *recA*) extracted from the *E. coli* MLST Database (University of Warwick) against each genome. Each allele is identified by a numeric marker. The combination of the 7 numeric markers for each strain was collected and used to obtain the Sequence Types (ST) (Table 1).

Pulsed-field gel electrophoresis

Strain clonality was checked by pulsed-field gel electrophoresis (PFGE) as described elsewhere (CDCPulseNetUSA, 2004). Agarose-embedded DNA was digested with 0.2 U/µl *XbaI* (Takara Bio) according to the manufacturer's instructions. The *XbaI*-digested genomic DNA was analysed on a 1% agarose gel in 0.5X Tris-boric acid-EDTA buffer at 14°C using the CHEF-DR III System (Bio-Rad). The gel was run for 19 h at 6 V/cm, with initial and final switch times of 2.2 s and 54.2 s, respectively. The gel was stained with ethidium bromide (1 µg/ml), and TIFF images were normalized and calibrated using GelComparII software (Applied Maths). Curve-based dendrograms were created using Pearson correlation

coefficients, applying 0.5% optimization and 0.5% of curve smoothing and the UPGMA clustering method. The dendrogram of the strains is shown in Supplementary Fig. 2.

Adhesion and invasion assays using Intestine-407 epithelial cells

The Intestine-407 epithelial cell line (ATCC CCL-6) was used for the adhesion and invasion assays. Cell culture, adhesion, and invasion assays were performed in triplicate as described previously[2]. Briefly, two 24-well plates containing $4x10^5$ cells/well that had been incubated for 20 h were infected at a multiplicity of infection of 10. Duplicate plates, one for the adhesion assay and one for the invasion assay, were incubated for 3 h at 37°C in 5% CO_2.

For the bacterial adhesion assays, the cell monolayers were washed five times with phosphate-buffered saline (PBS) and then lysed with 1% Triton X-100 (Sigma-Aldrich, St Louis, MO, USA). Adherent bacteria were quantified by plating them on Luria-Bertani (LB) agar (Liofilchem Srl, Italy). Plating was performed over a maximum period of 30 min to avoid bacterial lysis by Triton X-100. Adhesion ability (I_ADH) was determined by calculating the mean number of bacteria per cell. Isolates were considered adherent when I_ADH ≥ 1.

For the bacterial invasion assays, the monolayers were washed twice with PBS after 3 h of infection, and fresh cell culture medium containing 100 µg/ml gentamicin was added and left for 1 h to kill extracellular bacteria. After cell lysis with 1% Triton X-100, the number of intracellular bacteria was determined by plating. Invasive ability was expressed as the percentage of the initial inoculum that became intracellular: I_INV (%) = (intracellular bacteria/4x 10^6 bacteria inoculated) x 100. Isolates were considered invasive when I_INV ≥ 0.1%.

Host cell cytoskeleton involvement was evaluated as described by Baumgart *et al.*[3]. I-407 cells were seeded at a density of $4x10^5$ cells/well; after 24 hours, the monolayers were incubated with cytochalasin D (0.5 µg/ml) or colchicine (1 µg/ml) for 30 minutes to depolymerize microfilaments and microtubules, respectively. The monolayers were then

manipulated as described for the invasion assays. Finally, the inhibitory effect was determined and presented as the percentage of reduction of invasion indices.

Survival and replication within macrophages

The murine macrophage-like J774A.1 cell line (ATCC TIB-67) and the human THP-1 cell line (ATCC TIB-202) were used in survival and replication assays of the six sequenced *E. coli* strains. J774 cell culture was performed, and the ability of individual *E. coli* isolates to survive and replicate inside the macrophages was determined as described previously[4]. Briefly, J774 macrophages were seeded at $2x10^5$ cells per well in two 24-well plates. The plates were incubated for 20 h in complete medium (RPMI 1640 (Lonza, Switzerland) supplemented with 10% heat-inactivated FBS (Gibco BRL) and 1% L-glutamine (Gibco BRL)). After incubation, the medium was replaced with fresh medium, and bacteria were seeded at a multiplicity of infection of 10. To promote internalization of the bacteria by the macrophages, the plates were centrifuged at 900 rpm for 10 min and incubated for an additional 10 min at 37°C in 5% CO_2. Bacteria that were not phagocytosed were killed by inclusion of gentamicin (20 µg/ml) in the medium. The human THP-1 cell line (ATCC TIB-202) was maintained in RPMI 1640 medium (Lonza, Verviers, Belgium) supplemented with 10% (vol/vol) foetal bovine serum (Linus) in an atmosphere containing 5% CO_2 at 37°C. THP-1 cells were seeded in two 24-well plates at a density of $5x10^5$ cells per ml and were grown in complete medium containing 20 ng/ml of phorbol 12-myristate 13-acetate (PMA; Sigma-Aldrich) for 24 h to promote monocytic differentiation. After incubation, the medium was replaced with fresh medium (RPMI + 10% heat-inactivated FBS), and bacteria were seeded at a multiplicity of infection of 100. Similar to the J774 monolayers, the THP-1 plates were centrifuged at 900 rpm for 10 min and incubated for an additional 10 min at 37°C in 5% CO_2. The cell monolayers were washed twice with PBS, and fresh cell culture medium containing 100 µg gentamicin/ml was added to kill extracellular bacteria. After 40 min of incubation, one plate was washed twice with PBS, and 0.5 ml of 1% Triton X-100 (Sigma-Aldrich) was added to each well for 5 min to

lyse the eukaryotic cells. To determine the number of intracellular bacteria recovered, samples were diluted and plated onto LB agar plates. The medium of the second plate was replaced with fresh cell culture medium containing 20 µg/ml gentamicin and incubated for 23 h. Then, the monolayer was washed and treated with 1% Triton X-100, and the cell suspension was diluted and plated as described above.

For both cell lines, intracellular bacteria were quantified in the same manner as described for the invasion assays after 1 and 24 h of infection. The results are expressed as the mean percentage of bacteria recovered at 1 h and 24 h postinfection: I_REPL (%) = (CFU ml^{-1} at 24 h/CFU ml^{-1}at 1 h) * 100. Strains with an I_ADH higher than 1 bacterium/cell, I_INV of 0.1% and an I_REPL of 100% or higher were classified as AIEC strains in the present study.

DNA library preparation and sequencing

For Illumina sequencing, DNA samples were converted into sequencing libraries using the Illumina TruSeq DNA sample preparation kit at EA Quintiles. Briefly, 1 µg of genomic DNA was fragmented to ~200 bp using a Covaris E210 ultrasonicator. The fragmented DNA was then blunted, and a single “A-tail” was added to the 3’ end of each fragment to facilitate ligation of sequencing adapters containing a single T base overhang. The adapter-ligated DNA was amplified by the polymerase chain reaction to increase the amount of sequencing-ready DNA in the library. The final DNA libraries were analysed for size distribution and quality using an Agilent Bioanalyser (DNA 1000 kit, Agilent # 5067-1504), quantitated using Picogreen (Life Tech # P11496), and normalized to a concentration of 2 nM. Equal volumes of the normalized DNA libraries were pooled, and the pooled DNA was used to prepare a flow cell using the Illumina TruSeq Paired-End Cluster Kit V3 (Illumina # PE-401-3001). The pools were denatured using fresh 0.1 N NaOH and diluted to 20 pM in chilled hybridization buffer. The pools were further diluted to 9 pM, and an aliquot of each was placed in an Illumina cBot instrument to produce clusters through bridge amplification. Sequencing was

conducted on an Illumina HiSeq 2000 using 100-base paired-end sequencing plus a 7-base index cycle.

For the PacBio sequencing, the DNA libraries were prepared following PacBio guidelines and sequenced on SMRT cells using Pacific Biosciences RS sequencing technology (Pacific Biosciences, Menlo Park, CA, USA) at EA Quintiles. Ten micrograms of genomic DNA were purified using the PowerClean® DNA Clean-Up Kit (MO BIO Laboratories) and then sheared to 2 kb using a Covaris® Adaptive Focused Acoustics instrument. The sheared DNA was purified using magnetic beads and verified on a Bioanalyser. Library preparation was performed using the Pacific Biosciences DNA Template Prep Kit 2.0 (3 Kb - 10 Kb). Size selection and library purification were performed using 0.6X AMPure beads (Beckman-Coulter Genomics). Each library was bound to C2 DNA polymerase, loaded into a SMRT cell, and sequencing was observed using two 45-min movies for each cell. Quality analysis of the raw data was performed with PRINSEQ.

In silico **identification of strain-specific SNPs**

To determine the strain specificity of the Confirmed SNPs, we aligned the sequences containing the variable position in the six strains sequenced in this study as well as in 3 AIEC (UM146, LF82, NRG857c), 9 ExPEC (CFT073, 536, UMN026, S88, APEC01, 042, EDL933, O157 Sakaï, E24377A) and 3 commensal (HS, K12 MG1655, ED1a) strains and further determined the distribution of SNPs amongst the strains. We analysed the distribution within the strain collection of SNPs that displayed variability in the base under study among the strains and that occurred within genes that were widely distributed among the majority of the strains.

REFERENCES

1. Martinez-Medina, M. *et al.* Molecular diversity of *Escherichia coli* in the human gut: New ecological evidence supporting the role of adherent-invasive *E. coli* (AIEC) in

Crohn's disease. *Inflamm. Bowel Dis.* **15,** 872–882 (2009).

2. Boudeau, J., Glasser, A. L., Masseret, E., Joly, B. & Darfeuille-Michaud, A. Invasive ability of an *Escherichia coli* strain isolated from the ileal mucosa of a patient with Crohn's disease. *Infect. Immun.* **67,** 4499–509 (1999).

3. Baumgart, M. *et al.* Culture independent analysis of ileal mucosa reveals a selective increase in invasive *Escherichia coli* of novel phylogeny relative to depletion of Clostridiales in Crohn's disease involving the ileum. *ISME J.* **1,** 403–418 (2007).

4. Glasser, A. *et al.* Adherent invasive *Escherichia coli* strains from patients with Crohn's Disease survive and replicate within macrophages without inducing host cell death. *Infect. Immun. Immun* **69,** 5529–37 (2001).

5. Martinez-Medina, M. *et al.* Biofilm formation as a novel phenotypic feature of adherent-invasive *Escherichia coli* (AIEC). *BMC Microbiol.* **9,** 202 (2009).

SUPPLEMENTARY FIGURES

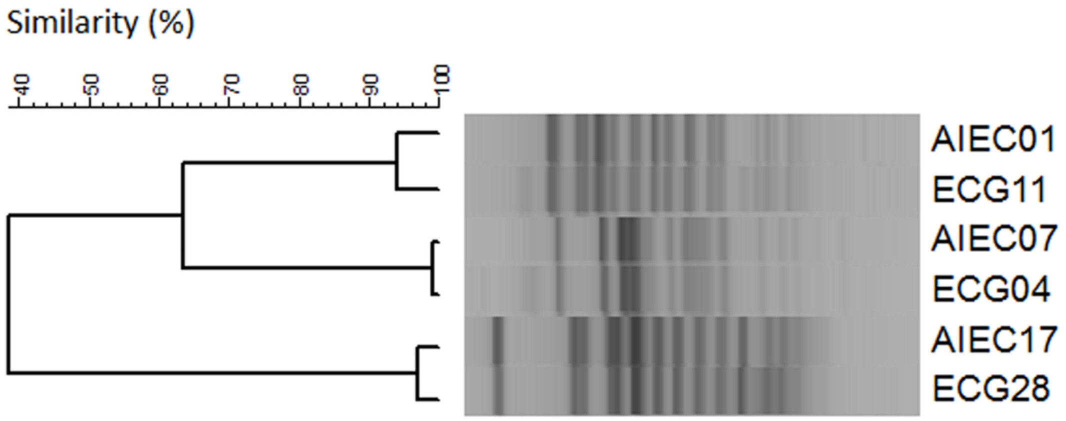

Figure S1. Consensus UPGMA dendrogram generated from the Pearson correlation coefficients of *XbaI* PFGE profiles of the three pairs of strains selected for genome sequencing. The bar indicates the percentage similarity of the profiles.

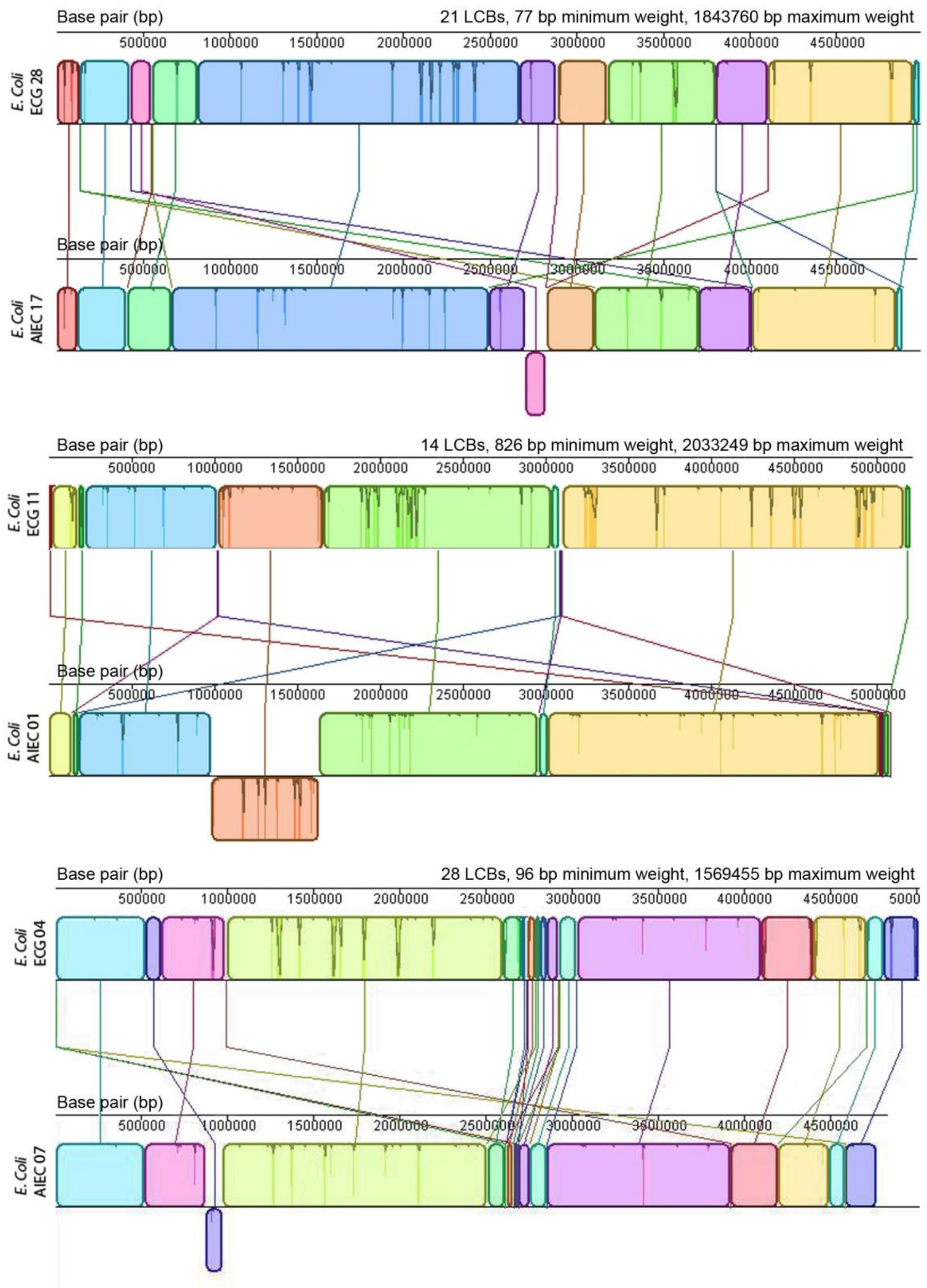

Figure S2. Whole genome map comparison of AIEC/non-AIEC strains prepared using MAUVE 2.3. Boxes of the same colour indicate homologous DNA segments between pairs. Breakpoints in the sequence are represented by the boundaries between the different coloured blocks.

SUPPLEMENTARY TABLES

Table S1. Assembly features of the AIEC/non-AIEC sequenced genomes.

Strain	Size (kb)	Contigs (No.)	GC (%)[a]	N50 (kb)[b]	Accession No.
AIEC17	4,958	400	50.42	247	ERS1456453
ECG28	4,981	464	50.38	214	ERS1456454
AIEC01	5,213	333	50.48	186	ERS1456455
ECG11[c]	5,212	25	50.56	555	ERS1456456
AIEC07	4,825	374	50.62	187	ERS1456457
ECG04	5,013	836	50.46	261	ERS1456458

[a] GC(%), content defined as (G+C)/(A+T+G+C)x100.

[b] N50, the length of the shortest contig at 50% of the assembly.

[c] This strain was sequenced by PacBio (library with 10kb insert) and assembled with HGAP 3 tool.

Table S2. Distribution of amino acid substitutions in three genes previously associated with AIEC pathogenesis in AIEC/non-AIEC strains. Amino acid substitutions that were previously associated with AIEC are marked in bold. The first letter corresponds to the amino acid present in the studied strain; the last letter indicates the amino acid found in the commensal strain K-12.

	Strains of study		
AIEC-associated genes	AIEC17-ECG28	AIEC01-ECG11	AIEC07-ECG04
fimH [23]	A27V, **S70N, N78S**	A27V, **H166R**	A27V, K32N*
ompA[25]	D46N, **V114I**, V196A, **T224N**, **A272G**	P46N, D47S, N48V, I49E, A125S, P128Y, GASF130-, D135N, V196A, **T224N**, **A272G**	P46N, D47S, N48V, I49E, **V114I**, A125S, P128Y, GASF130-, D135N, N172H, L182M,
chiA[26]	T100N, G166S, M182T, A200S, T286S, ETPV311, N326S, S335V, **Q362K, E370K, V378A, V388E**, M396L, I414V, N427D, T517A, **E548V**, A681D, R696K,S804S, Y810H, G811P	G166S, A200S, T286S, ETPV311-, N326A, S335V, **Q362K, E370K, V378A, V388E,** M396L, I414V, N416D, N427D, T517A, **E548V**, R696K	Absent

***Non-synonymous SNP reported in this study by comparative genomics, not associated with AIEC pathotype only present in AIEC07.

Table S3. Number of orthologous clusters of genes for each strain and specific clusters present in each strain compared to its counterpart.

AIEC17	ECG28	Shared	Only in AIEC17	Only in ECG28
4139	4137	4135	34661;D7XCN7-Cyanate transporter [b]	32006;B3Y1R0- Predicted protein[a]
			39495;G0FG36- TraR family protein[e]	36779;E6BSL1- Uncharacterized protein[a]
			57563;P64527-Uncharacterized protein YeeW[a]	
			59454-,Q46867-Inner membrane protein YgiZ[a]	

AIEC01	ECG11	Shared	Only in AIEC01	Only in ECG11
4293	4268	4260	3007;B1VC65-Hydrolase[e]	1036;Q8FCT4-Pimeloyl-[acyl-carrier protein] methyl ester esterase[e]
			25404;P0AG05-Streptomycin 3''-adenylyltransferase[e]	12287;C3SKM7-dTDP-glucose 4,6-dehydratase[e]
			30306;B1EKJ8-Uncharacterized protein[a]	23485;C3TC51-Aconitate hydratase[e]
			34057;D7XGD6-Uncharacterized protein[a]	24113;A0A137B781-Antitoxin HipB[e]
			36463;E7BTG4-Uncharacterized protein YubJ[a]	27497;A0A128B3E2-Putative AdoMet-dependent methyltransferase DLP12 prophage[e]
			41088;H4JWB9-Uncharacterized protein[a]	35556;Q47330-Putative glycosyltransferase[e]
			46848;I2B9P9-Putative inner membrane metabolite transport protein YdfJ[b]	97289;Q0Q086UTM4-Ascorbate-specific phosphotransferase enzyme IIB component[b]
			49208;J7QMD2-Uncharacterized protein, ECONIH1 strain[a]	116809;W8ZZ27- Uncharacterized protein[a]
			50771;I2B9X8-Histidine kinase[e]	
			51401;P19319-respiratory nitrate reductase 2[e]	
			55284;P03851-Uncharacterized 9.4 kDa protein[a]	
			55674;P0A908 -MltA-interacting protein[c]	
			55723;P0A9G4-HTH-type transcriptional regulator CueR[c]	
			56335;P0ACX1-Inner membrane protein YdgC[a]	
			56339;P0ACY5-Uncharacterized protein YeaG[a]	
			56474;P0AE32-Arginine ABC transporter permease protein ArtM[b]	
			56475;P0AE35-Arginine ABC transporter permease protein ArtQ[b]	
			57533;P24216-Flagellar hook-associated protein 2[d]	
			57640;P26608-Flagellar protein FliS[d]	
			57776;P30859-Putative ABC transporter arginine-binding protein 2[b]	
			57780;P30860-ABC transporter arginine-binding protein 1[b]	
			58516;P38105-Starvation-sensing protein RspB[c]	
			59044;P52108-Transcriptional regulatory protein RstA[c]	
			59768;P76097-Uncharacterized protein YdcJ[a]	
			59126;P56259-Putative uncharacterized protein YifN[a]	
			60232;P77400-Inner membrane transport protein YbaT[b]	
			60737;Q1R1V6-Uncharacterized protein[a]	
			61126; Q325B0-Glutaminase[e]	
			61671;Q57LR7-Cysteine synthase[e]	
			63434;Q9S4W7-Putative antirestriction protein YubI[a]	
			63745;C4ZQK4-Flagellar protein FliT[d]	

			63829;C4ZZI6-Protease HtpX[e]<br>64415;C4ZWZ4-UPF0060 membrane protein YnfA[a]	
**AIEC07**	**ECG04**	**Shared**	**Only in AIEC07**	**Only in ECG04**
4105	4121	4100	33786;E2QKQ2-Protein TonB[b]<br>37356;G0FCH4- Uncharacterized protein[a]<br>45186;I3RYC0- Uncharacterized protein[a]<br>45841;JSY690-Autotransporter UpaH[b]<br>58698;Q8VV25-*Escherichia coli* uropathogenic-specific protein[a]	24721;P0CF06-Insertion element IS1 2 protein InsA[e]<br>30689;B7JCB1- ABC-transport system, inner membrane component SitC[b]<br>31723;B7UPH0- Uncharacterized protein[a]<br>32043;C5J5A4-Fructose-like phosphotransferase enzyme IIB component 2[b]<br>32153;C8CGP6-Relaxosome protein TraM[e]<br>33090;D7X8P5- Gram-negative pili assembly chaperone domain protein[e]<br>34269;D9Z590-YfdA[a]<br>34690;E2QEX8-Antitoxin[e]<br>38492;G0FCH6-Uncharacterized protein [a]<br>38494;G0FCI7-Portal protein[e]<br>39795;H3MK58- Prepilin-type N-terminal cleavage/methylation domain-containing protein[e]<br>39833;H4I2H5-Uncharacterized Protein[a]<br>42792;H5V1F8-Putative Uncharacterized protein[a]<br>47160;J1QEW4-Integrase core domain protein[e]<br>50439;I2B5V7-Conserved barrel cupin 2 domain protein[e]<br>55545;P26841-HTH-type transcriptional regulator XapR[c]<br>57052;P45757-Putative type II secretion system protein C[b]<br>57299;P57018-Uncharacterized 9.0KDa protein[a]<br>59092;Q1RFR5- Uncharacterized protein[a]<br>59116;Q1RPL7-ECs1337 protein[a]<br>59330;Q46687-Fimbrial regulatory gene[e]

[a] Unknown function; [b] Related with transport; [c] Involved in transcriptional regulation; [d] Contribute to flagella assembly; [e] Other functions. BG7 annotation code and UniProt accession number are indicated for each gene and assigned according database July 2015.

Table S4. *E. coli* strain collection used for the validation of the suitability of SNP variants as putative signatures for AIEC detection. Phylogroup origin, type of patient from whom the strain was isolated (Crohn's disease (CD) or healthy subject (H)) and adhesion and invasion indices are indicated. Additional information about the strains characteristics can be found in Martinez-Medina, *et al*. (2009)[5].

Strains	Phylogroup	Phenotype	Origin	Adhesion index [a]	Invasion index [b]	Intramacrophage replication index in J774[c]
AIEC10	A	AIEC	H	5.9	0.226	1414
AIEC19	A	AIEC	H	2.4	0.111	1568
AIEC23	A	AIEC	CD	9.7	0.568	2362
AIEC24	A	AIEC	CD	2	0.309	1626
ECG16	A	Non-AIEC	H	0	0.002	na
ECG22	A	Non-AIEC	H	0	0.006	na
ECG18	A	Non-AIEC	CD	0.1	0.001	na
ECG19	A	Non-AIEC	CD	0	0.023	na
ECG65	A	Non-AIEC	CD	0.1	0.001	na
AIEC07	B1	AIEC	H	20	0.565	1693
ECG04	B1	Non-AIEC	H	0.5	0.023	813
ECG46	B1	Non-AIEC	H	0.0	0.001	na
ECG02	B1	Non-AIEC	CD	0	0.001	na
ECG21	B1	Non-AIEC	CD	6.1	0.033	na
ECG63	B1	Non-AIEC	CD	0	0.003	na
ECG64	B1	Non-AIEC	CD	0.3	0.019	na
AIEC04	B2	AIEC	H	21.6	0.32	585
AIEC06	B2	AIEC	H	10.2	0.177	1718
AIEC08	B2	AIEC	H	1.1	0.172	105
LF82	B2	AIEC	CD	25.7	2.261	777
AIEC01	B2	AIEC	CD	15.9	0.284	1567
AIEC02	B2	AIEC	CD	0.9	0.802	2188
AIEC05	B2	AIEC	CD	9.4	0.202	705
AIEC09	B2	AIEC	CD	5.4	0.216	2562
AIEC11	B2	AIEC	CD	4.4	0.508	848
AIEC12	B2	AIEC	CD	22.3	0.142	94
AIEC14	B2	AIEC	CD	9.8	0.238	801
AIEC15.1	B2	AIEC	CD	10	0.305	660
AIEC16.1	B2	AIEC	CD	9.7	1.400	921
AIEC21	B2	AIEC	CD	17	0.109	1297
AIEC25	B2	AIEC	CD	2.8	0.482	776
ECG08	B2	Non-AIEC	H	0.3	0.004	na
ECG12	B2	Non-AIEC	H	0.1	0.003	na
ECG13	B2	Non-AIEC	H	0.2	0.004	na
ECG17	B2	Non-AIEC	H	0.2	0.019	na
ECG41	B2	Non-AIEC	H	0.3	0.003	na
ECG43	B2	Non-AIEC	H	0.5	0.018	na
ECG49	B2	Non-AIEC	H	0.3	0.008	na
ECG01	B2	Non-AIEC	CD	0.2	0.002	na
ECG05	B2	Non-AIEC	CD	0	0.039	na
ECG09	B2	Non-AIEC	CD	0.2	0.001	na
ECG15	B2	Non-AIEC	CD	5.3	0.038	na
ECG26	B2	Non-AIEC	CD	0.2	0.005	na
ECG42	B2	Non-AIEC	CD	0	0.032	na
AIEC17	D	AIEC	CD	21.6	0.266	1053
AIEC20	D	AIEC	CD	14.2	0.125	344
ECG28	D	Non-AIEC	CD	2.2	0.057	1060
ECG34	D	Non-AIEC	CD	0.4	0.019	na

ECG57	D	Non-AIEC	CD	11.8	0.013	na
ECG23	Atypical	Non-AIEC	H	0.9	0.052	na

[a] Number of bacteria per I-407 cell. [b] Percentage of intracellular bacteria after 1h of gentamicin treatment relative to the inoculum. [c] Percentage of intracellular bacteria at 24h postinfection relative to the number after 1h of gentamicin treatment. na; Not Analysed.

Table S5. Information on the patients from whom the studied strains were isolated.

Id patient	Age	Gender	Disease status; origin of sample	Years since first diagnose	Treatment	Strains
53	33	M	C-CD; colon	nd	nd	ECG01, ECG02, AIEC25
55	42	F	CD, inactive; colon	12	mesalazine	ECG18
61	31	F	IC-CD, active; colon	0	none	ECG09, ECG65
69	67	M	Control (rectorrhagia, family history of colorectal cancer and polyposis); colon	na	na	ECG41
70	83	M	Control (diverticulosis); colon	na	na	ECG22
71	67	M	Control (micropolyps); colon	na	na	ECG12
72	27	F	C-CD, active; colon	0	none	ECG05, ECG64
77	48	F	I-CD, active; colon	1	6-mercaptopurine	AIEC21
79	25	F	I-CD, inactive; ileum	0	mesalazine	ECG19, ECG42
80	70	M	Control (constipation, irritable bowel syndrome); colon	na	na	ECG43
81	21	F	Control (diarrhoea and family history of colorectal cancer); colon	na	na	ECG46
82	29	F	Control (rectorrhagia); colon	na	na	ECG16
83	61	F	Control (rectorrhagia); colon	na	na	ECG17
84	59	F	Control (family history of colorectal cancer); colon	na	na	ECG49
89	31	M	IC-CD, active; colon	0	none	AIEC12
109	21	M	I-CD, inactive; colon	8	alternative medicine	ECG21, ECG34
110	37	F	IC-CD, inactive; colon	18	none	ECG63, ECG57, AIEC20
111	49	F	I-CD, inactive; colon	15	azathioprine	ECG28, AIEC17
112	35	F	C-CD, inactive; colon	19	azathioprine	ECG26
113	43	F	I-CD, inactive; colon	22	none	ECG15
114	31	F	IC-CD; colon	>2	nd	AIEC02, AIEC05
119	35	M	Control (rectorrhagia and family history of colorectal cancer); colon	na	na	ECG23, AIEC19
120	46	F	I-CD, inactive; ileum	13	azathioprine	ECG11 , AIEC01
122	31	M	IC-CD, inactive, perianal fistula; colon	10	azathioprine	AIEC09, AIEC24
123	36	M	C-CD, inactive; ileum	7	none	AIEC23
124	37	M	Control (rectorrhagia); ileum	na	na	ECG04, ECG08, AIEC07
125	33	F	Control (rectorrhagia); ileum	na	na	AIEC04
126	34	M	Control (rectorrhagia); ileum	na	na	AIEC10
127	30	M	Control (rectorrhagia); colon	na	na	ECG13
128	37	M	Control (constipation); colon	na	na	AIEC06
132	48	F	I-CD; ileum	9	nd	AIEC11, AIEC14, AIEC15.1, AIEC16.1
142	41	M	Control; colon	na	na	AIEC08

M: Male; F: Female; na: not applicable; nd: no data; C-CD: colonic Crohn's disease; I-CD: ileal Crohn's disease; IC-CD: ileocolonic Crohn's disease. Active when CDAI>150. LF82 strain was also studied[2].

Table S6. Primers and PCR conditions used to amplify fragments of the genes in which the Confirmed SNPs were located.

Gene ID	Primer Forward	Primer Reverse	Annealing temperature (ºC)
E1-E2_3.4	TCCTCAATGAATCGCAGTCTC	TCAAAAGATTGCCCGCTTAC	57
E1-E2_3.6	CTCATCAGCCGGACATACG	CACCTGTTTTCACTTTTATCTTCTG	56
E1-E2_3.7	GGTAACCCATTTGGCCTTG	CAACACTTCGCTGACAAACG	57
E1-E2_5	CGCTATAACGGCGAACTGAT	TCAGTGGTCCGGTATCAAAA	56
E3-E4_4.2	GCCAGTAACTCTTCGCCATT	TCAGGACAGCGACAAAAGC	57
E3-E4_4.3	GTTTTCTCCTTTGCCGAACA	TGATGGTGATAATGCTGCTCA	57
E3-E4_4.4	ATATTCAGCCTGTCCGCAAT	CGCATCATCACTTCCATCTG	57
E3-E4_4.5	GCGTTGCCTGATGATACTGA	CGTCGGGGACATCTGACTTA	57
E3-E4_4.7	GGAAGAGCTGGAGACAATGC	CACTACCGCCACTCTCCTGT	57
E5-E6_3.1	CCCTGTTTGCTGTACTGCTG	CTGCTCACAGGCGTCAAATA	56
E5-E6_3.12	GAAAAAGTCGCCCATGAGAC	CGCAACACCAGAGGGTTAAT	57
E5-E6_3.16=3.22	CATCACTTCCGGTCAGCAC	ATTGCAGAAAAGCGAGAGGT	56
E5-E6_3.17	TTTTCACWCGAAGGTCGATG	GATGTGCTGCTGTGCTGYTT	56

PCR program: 1 cycle at 95ºC for 5 minutes, 30 cycles of 15 seconds at 95ºC and 45 seconds at the primer annealing temperature, finally, one cycle at 72ºC during 10 minutes. All primers were used at 0.2µM; PCR Buffer II at 1x; $MgCl_2$ at 1.5mM; dNTPs at 200µM and AmpliTaq Gold polymerase 1.25units/reaction.
